# A Narrative Review of Post-traumatic Neuroinflammation: Relevance to Pediatrics

**DOI:** 10.7759/cureus.69512

**Published:** 2024-09-16

**Authors:** Ankita Patel, Amar Taksande, Rahul Khandelwal, Aditya Jain

**Affiliations:** 1 Pediatrics, Jawaharlal Nehru Medical College, Datta Meghe Institute of Higher Education and Research, Wardha, IND

**Keywords:** neuroinflammatory response, pediatric neuroinflammation, pediatric neurology, pediatric traumatic brain injury, post-traumatic neuroinflammation, traumatic brain injury

## Abstract

This is a narrative review that explores the complex interaction between post-traumatic neuroinflammation and its importance in pediatric traumatic brain injuries (TBIs). For immediate and long-term consequences of TBI, neuroinflammation, manifested by activation of microglia and astrocytes, secretion of pro-inflammatory cytokines, as well as breakdown of the blood-brain barrier, are critical factors. While inflammation is an essential part of the brain’s repair systems, excessive or prolonged neuroinflammation can lead to more significant neuronal damage, which, in turn, causes persistent cognitive and behavioral deficits over time. In this regard, the paper synthesizes existing evidence concerning molecular and cellular mechanisms that underlie neuroinflammation in relation to TBI among children, paying attention to age disparities in inflammatory response and their implications for treatment and recovery. Furthermore, it explores how targeted anti-inflammatory therapies are highly likely to improve outcomes for pediatric patients. The outcomes emphasize that there is a need for a more comprehensive understanding of child neuroinflammatory processes and age-specific therapeutic approaches aimed at lessening the effects’ negative impacts after brain injury occurs.

## Introduction and background

Post-traumatic neuroinflammation (PTNI), characterized by the activation of the central nervous system's (CNS) immune response following traumatic brain injury (TBI), has emerged as a critical factor influencing the long-term outcomes of brain injuries, particularly in the pediatric population. The developing brain is uniquely vulnerable to injury, and the inflammatory response triggered by trauma can lead to a cascade of secondary injury mechanisms that exacerbate neuronal damage and impair recovery [1]. In children, the consequences of neuroinflammation are particularly concerning due to the ongoing brain development and the potential for long-lasting effects on cognitive, emotional, and motor functions [2]. Research has shown that the pediatric brain responds differently to trauma compared to the adult brain, with variations in inflammatory mediator expression, BBB permeability, and neurogenesis [3]. It is crucial to understand the differences between PTNI in children, due to its uniqueness, for the sake of developing age-specific therapeutic strategies. Although there is increasing awareness regarding the role of neuroinflammation in pediatric TBI, few comprehensive reviews exist that summarize what is known about this intricate process and how it can be managed clinically in cases involving children.

This narrative review aims to fill this gap by providing an overview of the current literature on PTNI in the pediatric population, highlighting the underlying mechanisms, potential biomarkers, and therapeutic approaches. By focusing on the pediatric population, this review seeks to elucidate the relevance of neuroinflammation in shaping the outcomes of TBI in children and to identify areas where further research is needed to improve treatment and prognosis.

## Review

Search methodology

To conduct a narrative review on "Post-traumatic Neuroinflammation: Relevance to Pediatrics," a comprehensive search was executed across multiple scientific databases, including PubMed, Scopus, and Web of Science, up to the current date. This involved combining keywords and MeSH terms such as "post-traumatic neuroinflammation," "neuroinflammation," "pediatric brain injury," and "traumatic brain injury in children." The studies that were eligible for inclusion consisted of original research articles, systematic reviews, and clinical guidelines that look at the mechanisms, outcomes, and management of neuroinflammation that follows traumatic head injury in young people. Exclusion criteria include non-English language articles, research not concerned with people under 18, and those lacking pertinent information on neuroinflammation. Reference lists from selected studies were searched manually for more relevant studies. Data extraction will emphasize understanding the mechanisms behind neuroinflammation, its clinical effects in infants or children, and possible treatments for this condition. Therefore, the synthesized findings would provide a complete outlook on what we currently know about PTNI for pediatric care today.

Pathophysiology of neuroinflammation

Mechanisms of Neuroinflammation

Neuroinflammation is a complicated response that can manifest in many different ways, depending on the various types of CNS injury sustained, such as TBI. BBB disruption is common after TBI, letting peripheral immune cells and molecules into the CNS, where they maintain a pro-inflammatory environment. The main mechanisms include activating resident CNS immune cells, like microglia and astrocytes, which respond to trauma by producing pro-inflammatory chemokines, cytokines, and reactive oxygen species (ROS). This protective inflammatory response may become dysregulated, leading to secondary neuronal damage and further aggravating neuroinflammation [4].

Role of Microglia and Astrocytes

Microglia, the resident macrophages of the CNS, function crucially in starting and keeping neuroinflammation in check. When signals from wounds make them active, microglia change in their morphology and function as they become activated - producing inflammatory substances, consuming cellular waste, and modifying the local immune setting [5]. Astrocytes, the most abundant glial cells in the CNS, also contribute to neuroinflammation by releasing cytokines, chemokines, and growth factors. In the context of TBI, astrocytes can form a glial scar around the injury site, which can both protect surrounding tissue and limit neural regeneration [6]. Together, microglia and astrocytes orchestrate a complex interplay of inflammatory signals that can exacerbate neuronal injury if not properly regulated.

Cytokine Release and Inflammatory Cascade

**Figure 1:**
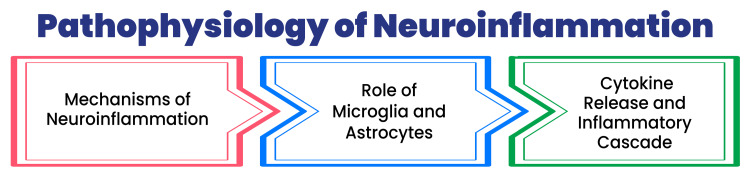
Pathophysiology of neuroinflammation Image credits: Dr. Ankita Patel

The release of cytokines is a hallmark of the neuroinflammatory response following TBI. Pro-inflammatory cytokines, such as interleukin-1β (IL-1β), tumour necrosis factor-alpha (TNF-α), and IL-6, are rapidly produced by activated microglia and astrocytes and contribute to the amplification of the inflammatory cascade [7]. These cytokines promote adhesion molecule expression in endothelial cells, enabling peripheral immune cells to access the CNS. At the same time, they stimulate the production of other inflammatory mediators, like ROS and nitric oxide (NO), that may worsen neuronal injury. Moreover, the persistent inflammatory cascade leads to chronic neuroinflammation, which is associated with lasting neurological deficits, particularly in children whose brains are still developing [8]. Figure 1 shows the pathophysiology of neuroinflammation.

TBI in pediatrics

Epidemiology of Pediatric TBI

TBI is a significant cause of morbidity and mortality in the pediatric population. In the United States, approximately 500,000 children are treated for TBI annually, with falls, motor vehicle accidents, and sports-related injuries being the most common causes [9]. Younger children, particularly those under the age of four, are at a higher risk for TBI due to their developing motor skills and increased likelihood of falls [10]. The incidence of TBI is also higher in adolescents due to risk-taking behaviors and sports participation [11].

Unique Vulnerabilities in the Pediatric Brain

The pediatric brain is uniquely vulnerable to traumatic injury due to several factors. The brain is still in a critical development phase, with ongoing processes such as synaptogenesis, myelination, and cortical maturation, making it more susceptible to injury. Additionally, the pediatric brain is more likely to recover than the adult brain [12]. The pediatric skull is thinner and more pliable, providing less protection against external forces [13]. The higher water content and incomplete myelination of the pediatric brain further contribute to its increased vulnerability to mechanical forces during trauma [14].

Short-Term and Long-Term Consequences of TBI

The consequences of pediatric TBI can be profound and long-lasting. In the short term, children may experience a range of symptoms, including headaches, vomiting, loss of consciousness, and seizures [15]. Cognitive impairments, behavioral changes, and emotional disturbances are also common in the acute phase following TBI [16]. Long-term consequences may include persistent cognitive deficits, particularly in attention, memory, and executive function [17]. There is also an increased risk of psychiatric disorders, including depression and anxiety, as well as difficulties in academic and social functioning as the child grows [18]. Furthermore, repeated TBIs can exacerbate these effects and lead to more severe neurodevelopmental outcomes [19].

PTNI in pediatrics

Acute Phase of Neuroinflammation

In the case of the acute neuroinflammatory process after TBI in children, there is an immediate and strong immune response characterizing it. Microglia and astrocytes in place are activated, aiming to release pro-inflammatory cytokines, chemokines, and ROS. The purpose of this primary action is to limit destruction and enhance tissue healing, but it may also bring about more neuronal death through mechanisms such as excitotoxicity or BBB breakdown [20]. In the pediatric brain, which is still developing, the acute inflammatory response may be particularly harmful, as it can interfere with critical periods of neurodevelopment, potentially leading to long-term cognitive and behavioral deficits [21].

Chronic Neuroinflammation

Inflammation that lasts for a long time is called chronic neuroinflammation, a prolonged inflammatory reaction that persists long after the initial injury. This condition often leads to neurodegeneration and functional impairments. In pediatric TBI, activated microglia and astrocytes can result in the continuous release of inflammatory modulators, creating a cycle of inflammation that maintains neural damage. This persistent inflammation has been associated with various long-term outcomes, such as neurodegenerative disorders as well as psychiatric diseases [22]. The pediatric population is particularly vulnerable to the effects of chronic neuroinflammation due to the ongoing maturation of the brain, which can be disrupted by prolonged exposure to inflammatory cytokines, leading to abnormal synaptic pruning, myelination deficits, and altered neuroplasticity [23].

Impact on Neurodevelopment

Neurodevelopment in children is profoundly affected by neuroinflammation following trauma to the brain because these inflammatory processes can disrupt important developmental phases. During these critical periods, the brain grows significantly and undergoes major changes, such as altered neuronal connections, synaptic remodeling, and cognitive dysfunction due to neuroinflammation. It has been documented that children who suffer from TBI are at an increased risk for developmental delays, learning difficulties, and conduct problems that are frequently correlated with post-injury neuroinflammatory responses [13]. Moreover, the interplay between neuroinflammation and neurodevelopmental processes may increase susceptibility to neuropsychiatric disorders, such as attention deficit hyperactivity disorder (ADHD), anxiety, and depression later in life, underscoring the importance of early intervention and therapeutic strategies aimed at mitigating inflammation in pediatric TBI [24].

Diagnostic challenges

Identifying Neuroinflammation in Pediatrics

Diagnosing neuroinflammation in children presents unique challenges due to how subtle and varied symptoms can be in younger people. Neuroinflammation can have broad clinical implications, making it hard to diagnose since symptoms may resemble those of other disorders. For example, children who suffer from TBI may show irritability, cognitive delays, or behavioral changes, and these symptoms may not have any other apparent cause, enabling the syndrome to be attributed to many post-trauma sequelae [25]. Moreover, developmental changes in the brain can influence how neuroinflammation manifests, making it difficult to establish clear diagnostic criteria. Pediatric patients may also be less able to articulate symptoms, further complicating the clinical assessment [26].

Biomarkers for Neuroinflammation

Finding reliable biomarkers for neuroinflammation in pediatric patients can be a significant challenge. Some cytokines and proteins involved in inflammation have been found to act as markers showing promise; however, they have not yet been standardized for clinical use. For instance, studies have reported increased inflammatory markers, such as IL-6 and TNF-α, in children with TBI. Still, these results are not consistent everywhere due to various factors, including age and injury severity [27]. Additionally, the complexity of biomarker interactions and the lack of age-specific reference ranges complicate the interpretation of these biomarkers in the pediatric population [28].

Imaging Techniques

Imaging techniques to diagnose neuroinflammation in pediatric patients involve several challenges. While magnetic resonance imaging (MRI) and positron emission tomography (PET) are valuable tools, their application in children is complicated by issues such as the need for sedation, patient movement, and the high sensitivity of these techniques to artifacts [29]. Although MRI can detect structural neuroinflammation-related changes, it does not always highlight early or subtle inflammatory changes. On the other hand, PET imaging provides functional insights, although it is limited by radiation exposure and accessibility [30]. Thus, integrating advanced imaging techniques with clinical assessments is crucial for a comprehensive evaluation of neuroinflammation in pediatric cases.

Management and therapeutic approaches

Current Treatment Strategies

The technique for dealing with PTNI in children depends on many factors, including the use of medications and natural approaches. Most actions being engaged aim to assist in symptomatic control and avoid additional neuronal harm. Generally accepted immediate care, especially at this stage, consists, for example, of addressing intracranial pressure and ensuring effective blood flow to the brain. Furthermore, measures to minimize secondary injuries, such as those caused by seizures or metabolic disorders, are also important [31]. Rehabilitation efforts, including physical, occupational, and cognitive therapy, are integral for long-term recovery and functional outcomes [32].

Anti-inflammatory Treatments

Treatments for anti-inflammatory purposes are key to handling PTNI since they aim at the inflammatory pathway which happens after head trauma. One of the most popular of these is dexamethasone, a class of medications called corticosteroids that are often administered to decrease irritation and swelling within the brain. Nevertheless, there is still no clear answer regarding its use in children because they may have undesirable effects or differences in effectiveness [33]. More recent studies have explored using non-steroidal anti-inflammatory drugs (NSAIDs) and selective cytokine inhibitors, such as IL-1 receptor antagonists, to modulate the inflammatory response more precisely [34]. While these treatments show promise, their safety and effectiveness in children require further investigation.

Emerging Therapies

Emerging PTNI therapies in pediatric patients focus on innovative approaches to modulate the inflammatory response and promote neuroprotection. Advances in gene therapy, including gene-editing tools like CRISPR/Cas9, hold the potential for directly targeting inflammatory pathways and enhancing neuronal resilience [34]. Additionally, stem cell therapy is being explored for its ability to repair damaged neural tissue and reduce inflammation [35]. These therapies are in various stages of clinical research, and, while preliminary results are promising, further studies are needed to establish their safety and efficacy in pediatric populations.

Clinical implications and future directions

The narrative review on PTNI highlights critical implications for pediatric care and identifies several avenues for future research. The authors emphasize the need for early and accurate diagnosis of neuroinflammation after TBI; this is important because timely management can help avoid cognitive or neurological deficits over time. Pediatricians and neurologists should always be prepared to recognize potential symptoms of neuroinflammation in young people with a history of trauma, as this is a crucial step in determining the appropriate use of advanced imaging techniques and biomarkers.

Future research should focus on longitudinal studies to better understand the long-term effects of PTNI on cognitive and behavioral outcomes in pediatric populations. Investigating novel therapeutic interventions, such as anti-inflammatory agents or neuroprotective strategies, could offer new avenues for treatment and prevention. Additionally, exploring the genetic and molecular mechanisms underlying individual variability in neuroinflammatory responses may help tailor more personalized approaches to managing and mitigating the effects of TBI in children. Collaborative research efforts and the development of standardized protocols for assessing and managing PTNI will be crucial in improving patient outcomes and advancing the field.

## Conclusions

In conclusion, this narrative review highlights the critical role of PTNI in the pediatric population, emphasizing its significant impact on long-term neurological outcomes. Despite advances in understanding the underlying mechanisms, the variability in study methodologies and outcomes underscores the need for further research to clarify the precise pathways and develop targeted therapeutic strategies. Early detection and intervention remain pivotal, as they may mitigate the adverse effects of neuroinflammation and improve recovery trajectories. Future research should focus on refining diagnostic tools, exploring novel treatment options, and establishing long-term follow-up protocols to better manage and prevent PTNI in pediatric patients.
